# Meaningful changes in physical function and pain in patients with knee osteoarthritis

**DOI:** 10.1186/s41687-025-00941-x

**Published:** 2025-09-26

**Authors:** Xiaodan Tang, Jin-Shei Lai, Sung Keun Kang, John Devin Peipert

**Affiliations:** 1 Department of Medical Social Sciences, Northwestern University Feinberg School of Medicine, Suite 2700, 625 N Michigan Ave, Chicago, IL 60611 USA; 2 Biostar Stem Cell Research Institute, RBio Co., Ltd, Seoul, Korea; 3 Department of Applied Health Sciences, University of Birmingham, Birmingham, UK

**Keywords:** Meaningful change, WOMAC, Physical function, Pain, Treatment effectiveness, Knee osteoarthritis

## Abstract

**Background:**

This study aims to establish meaningful within-person change (MWPC) thresholds for the Total Western Ontario and McMaster Universities Osteoarthritis Index (WOMAC^®^), its Physical Function (PF) subscale, and the Visual Analog Scale (VAS) for Pain, in patients with knee osteoarthritis (OA). A secondary objective is to evaluate the effectiveness of a single injection of autologous culture-expanded adipose tissue-derived mesenchymal stem cells (ADMSCs) using these thresholds.

**Methodology:**

The study included 252 patients with knee OA enrolled in a clinical trial. An anchor-based predictive modeling approach, using KOOS-12, SF-36, and IKDC scores with literature-based cut-offs, was applied to determine MWPC thresholds for WOMAC and VAS Pain. MWPC thresholds were derived from both the ADMSCs injection and Control (autoserum) groups at 3- and 6-month follow-ups. Treatment effectiveness was assessed by comparing MWPC range with between-group differences and within-person changes.

**Results:**

MWPC thresholds were identified as follows: 5–17 points for WOMAC Total, 4–12 points for WOMAC PF, and 8–14 points for VAS Pain (all on 0–100 scales). The adjusted differences between the ADMSCs injection and Control (autoserum) groups of all three measures (3 months: WOMAC Total 5.9, WOMAC Function 4.2, VAS Pain 8.6; 6 months: WOMAC Total 9.3, WOMAC Function 6.5, VAS Pain 11.7) were within each corresponding MWPC threshold range.

**Conclusions:**

The proposed MWPC thresholds could be beneficial for healthcare professionals as a tool to identify meaningful change in physical function and pain in response to treatment and evaluate the meaningfulness of treatment benefits.

**Supplementary Information:**

The online version contains supplementary material available at 10.1186/s41687-025-00941-x.

## Background

Osteoarthritis (OA) is among the most prevalent musculoskeletal conditions globally [1]. Pain and worsened physical function significantly contribute to the disability associated with OA, making the improvement of pain and physical function a frequent treatment goal [2, 3]. Treatment benefits are often evaluated using patient-reported outcome measures (PROMs) [4, 5], such as the Western Ontario and McMaster Universities Osteoarthritis Index (WOMAC^®^) [6, 7] and visual analog scale (VAS) [8]. WOMAC is a validated PROM designed for patients with hip or knee OA with subscales of Pain, Stiffness, and Physical Function [6, 7]. The 100-mm VAS is also widely used to assess pain severity [9]. 

Although the approach of determining whether individual changes are statistically significant (i.e., minimal detectable change and responder definitions) is sometimes used to assess new therapies and conduct comparative effectiveness research, it is more crucial to demonstrate meaningful change, a change in patients’ outcomes that is noticeable and important from patients’ perspective, to confirm the efficacy of new treatments [5, 10]. Patients typically view meaningful change as a real-world difference in their health-related quality of life. This is consistent with the suggestion by Vickers et al. (2025) [11] that the term “minimal” should be avoided when describing important differences, as their interpretation should depend on the specific treatment context rather than a universal threshold. Additionally, the US Food and Drug Administration (FDA) has published guidelines [12] emphasizing the importance of defining meaningful within-patient change (MWPC) from the patient’s perspective and establishing thresholds to support the clinical interpretation of treatment benefits. Among several methods to calculate MWPC, the anchor-based approach has been commonly used because it uses an external criterion (i.e., anchor), such as patients’ global rating of change or certain clinical marker, that provides an independent, interpretable reference point to assess whether the change in a patient-reported outcome is meaningful.

There are several methods to estimate the MWPC thresholds and their range using the anchor-based approach: the mean change method, receiver operating characteristics (ROC), predictive modeling, and vignettes. No single gold standard method is currently recommended in clinical significance studies. In general, the mean change method is more commonly used than the other two methods [13]. However, Terwee et al. [14] suggested that the predictive modeling method is the most appropriate method because it directly calculates a threshold for meaningful change, while the mean change method calculates the mean of change scores to represent the thresholds. Additionally, it avoids simple mean differences, which can be biased by distributional imbalances or sample heterogeneity. Further, predictive modeling is more precise than the ROC approach and can be corrected for bias if the prevalence of the change group is not 50%, and is therefore recommended as the best option [14].

A few studies have examined the MWPC thresholds for WOMAC and VAS pain. Angst et al. found the difference in mean change scores in WOMAC between the “slightly better” and the “equal” groups was 0.80 in WOMAC PF and 0.82 in WOMAC Total on a 0–10 scale [15]. In another study, the proposed thresholds were 19.9 for VAS Pain and 9.1 for WOMAC PF on a 0-100 scale calculated by using the change score at the 75th percentile among those who rated their intervention as ‘good, satisfactory effect with occasional episodes of pain or stiffness’ [5]. A third study reported the MWPC thresholds of WOMAC PF as 0.85–1.14 (on a 0–10 scale) for 1-category change on the one-item patient global impression of disease severity scale, and 1.70–2.28 for 2-category change [10]. All these studies relied on the means or percentiles of the observed change scores. There is a lack of studies applying the innovative and promising predictive modeling approach to calculate MWPC values for WOMAC and VAS Pain. Without the predictive modeling approach, clinicians and researchers may misclassify treatment responders or underestimate meaningful change, potentially limiting the precision of patient-centered care and the accurate evaluation of treatment benefits. Addressing this gap is essential to improve the interpretability and clinical relevance of WOMAC and VAS pain in knee OA.

This study aims to use the predictive modeling method to determine the range of MWPC thresholds for the total WOMAC score (denoted as WOMAC Total), the WOMAC physical function subscale (denoted as WOMAC PF) and the VAS Pain subscale (denoted as VAS Pain) in patients with knee OA. Further, we aim to use these MWPC thresholds to evaluate the meaningfulness of treatment benefits for the single injection of autologous culture-expanded adipose tissue-derived mesenchymal stem cells (ADMSCs) treatment.

## Materials and methods

### Data

The analysis data of this study was approved by the institutional review board of Kyung-Hee University Hospital at Gangdong (KHNMC 2019-04-017-001) and registered at ClinicalTrials.gov (NCT03990805) before enrollment of the first patient [16]. This double-blind randomized controlled trial enrolled 261 patients with K-L grade 3 symptomatic knee OA, of whom 252 received a single injection of either autologous culture-expanded ADMSCs (*N* = 125) or Control (autoserum) (*N* = 127). Informed consent was obtained from all patients. Clinical and PROM data were assessed at baseline, 3 months, and 6 months after the injection. Among all participants, 246 (124 in the ADMSCs injection group, 122 in the control/autoserum group) completed assessment at 3 months, 247 at 6 months (124 in the ADMSCs injection group, 123 in the Control/autoserum group). None of the variables analyzed in this study contain missing data. The primary endpoints of this trial were improvements in 100-mm VAS Pain, WOMAC Total, and WOMAC PF at 6 months. Additional PROMs included the Knee Injury and OA Outcome Score (KOOS) [17] the SF (Short Form)-36 Health Survey (SF-36) [18] and the International Knee Documentation Committee (IKDC) Subjective Knee Form [19] which were used as anchors to derive MWPC thresholds in this study. Demographics and baseline characteristics have been published in another paper [16]. 

## Measures

### Focal patient-reported outcome measures

#### WOMAC total and physical function subscale

WOMAC measure was developed in 1982 at Western Ontario and McMaster Universities, which has been widely used in the evaluation of Hip and Knee OA [6]. It is a self-administered questionnaire consisting of 24 items divided into 3 subscales: Pain (5 items), Stiffness (2 items) and Physical Function (17 items). The total score and subscale scores are converted to a score out of 100. Higher total scores on the WOMAC indicate worse pain, stiffness, and more physical functional limitations.

#### VAS pain

The VAS Pain rating scale was used for the first time in 1921 by Hayes and Patterson [8]. In this study, VAS Pain was assessed using a straight horizontal line of fixed length 100-mm. The ends are defined as the extreme limits of pain orientated from the left (best) to the right (worst). The VAS Pain score is determined by measuring in millimeters from the left-hand end of the line to the point that the patient marks.

### Anchor measures

We applied the following criteria to select the anchor measures according to the FDA guidance [12]: (1) patient-reported; (2) function-targeted or pain-targeted; (3) plainly understood in the clinical context of OA and easier to interpret than WOMAC Total, WOMAC PF and VAS Pain; (4) the correlation between the change scores of WOMAC Total, WOMAC PF, VAS Pain and the anchors should be at least 0.371 [20], and ideally higher than 0.50 to assume validity of being used as anchors [21]. Based on these criteria, we selected the measures of KOOS-12 (pain and function subscales), SF-36 (physical function subscale), and IKDC (total scores) as candidates. Correlation analyses confirmed that all selected anchors had correlations above 0.45 with WOMAC and VAS Pain change scores.

#### IKDC

The International Knee Documentation Committee (IKDC) Questionnaire is a knee-specific patient-reported outcome measure and a subjective scale that provides an overall function score [19]. It consists of 3 categories: symptoms, sports activity, and knee function, and 19 items. The IKDC total score was calculated based on a 0-100 metric. Higher scores indicate higher levels of function and lower levels of symptoms.

#### KOOS-12

KOOS is a 42-item self-administered knee-specific assessment of five outcomes: knee-related quality of life, activities of daily living, sport and recreation function, symptoms, stiffness and pain [17]. Each item is scored from 0 (no problems) to 4 (extreme problems). Based on the KOOS measure, a 12-item short form of the Knee injury and OA Outcome Score was developed [22]. It consists of pain, function, quality of life subscales and four items in each subscale. Each KOOS-12 subscale score as well as the scale score is calculated by summing up all question responses in the subscale and transformed to a 0-100 scale so 0 is the worst possible and 100 is the best possible score. Higher KOOS-12 scores indicate better condition.

#### SF-36

SF-36 is a multi-dimensional PROM frequently used across multiple areas of medicine [18]. It comprises 36 questions that cover eight domains of health. We will use the 10-item physical function subscale as an anchor in this study. The raw scores are converted and pooled using a scoring key, for a total score indicating a range of low to high quality of life [23]. Higher score would indicate better condition.

### Statistical analyses

We first identified the improvement categories for each anchor by reviewing the literature [24–26] to find their mean change scores corresponding to each improvement level of the Patient Global Impression of Change (PGIC), an anchor measure commonly used in clinical trials due to its conceptual clarity. These mean change scores and correlations with the focal measures are provided in Appendix 1. Based on these scores, we derived thresholds by calculating the mean between the first and third levels of improvement on the PGIC and rounding to the nearest integer to define meaningful improvement. This would indicate meaningful improvement for patients while ensuring they are not set too high, making them difficult to achieve. The specific thresholds we used are: the KOOS-12 Total scores improved between 2 and 15 points [24], KOOS-12 Function improved 2 to 15 points [24], KOOS-12 Pain improved 2 to 17 points [24], IKDC improved between 7 and 19 points [25], and SF-36 Physical summary score improved 1 to 5 points [26]. According to the content relevance, we used KOOS-12 Total and IKDC as anchors to calculate MWPC values for WOMAC Total; KOOS-12 Function, IKDC, and SF-36 Physical for WOMAC PF; and KOOS-12 Pain for VAS Pain. We did not derive thresholds for worsening due to the small number of participants with worsened scores.

Based on these anchor change categories, we applied the predictive modeling approach [14, 27] to identify the MWPC values of WOMAC Total, WOMAC PF, and VAS Pain at both 3-month and 6-month time points and separately for the ADMSCs injection and Control (autoserum) groups. The predictive modeling approach fits a logistic regression model with anchors classified as improved vs. not improved as the dependent variable and WOMAC Total, WOMAC PF or VAS Pain change scores as the independent variable. The MWPC estimate was calculated by taking the WOMAC Total, WOMAC PF or VAS Pain change score value associated with a likelihood ratio of 1, which provides a probability-based threshold between improved and not improved patients. To generate the 95% confidence intervals of the calculated thresholds, we used nonparametric bootstrapping based on 1,000 simulated samples. We also adjusted for the unequal prevalence of the improved groups. The overall approach to identifying the range of MWPC thresholds is to compile all the MWPC values calculated from both the ADMSCs injection and Control (autoserum) groups at the 3-month and 6-month time points.

We then evaluated the meaningfulness of the benefits from the ADMSCs injection treatment using the derived MWPC thresholds. First, we built ANCOVA models controlling for baseline scores and calculated the least-squares group means of each group after controlling for baseline scores at both time points. The between-group differences were then compared against the proposed range of the MWPC thresholds. Second, we ran another set of ANCOVA models on the within-person changes between the two groups controlling for baseline scores. We then compared them to the range of the MWPC thresholds.

## Results

As shown in Table 1, we calculated the MWPC values for improvement based on each anchor change category for WOMAC Total, WOMAC PF, and VAS Pain, stratified by time point (3 and 6 months) and treatment group (ADMSC injection and control). The adjusted MWPC thresholds were generally higher at 6 months than at 3 months, with WOMAC PF thresholds consistently lower than those for WOMAC Total. The Control (autoserum) group tended to have lower MWPC values than the ADMSCs injection group. Across all focal PROs, the MWPC values derived from multiple anchors did not differ substantially from one another and were consistent, indicating they were within a reasonable range. We combined MWPC values calculated from both groups and time points, identified minimum and maximum MWPC values, and rounded them to the nearest integers to define the range for each measure. We then proposed a range of 5 to 17 score points as MWPC thresholds for improvement in WOMAC Total, a range of 4 to 12 score points for improvement in WOMAC PF, and a range of 8 to 14 score points for improvement in VAS Pain.

**Table 1:** MWPC values for WOMAC total, WOMAC function, and VAS pain

Measure	Group	Time	Anchor	Category	*N*	Adjusted Meaningful Within-Person Change	Confidence Interval
WOMAC Total	ADMSCs injection	3 months	KOOS-12 Total	Meaningful improvement (≥ 2&≤15)	50	-10.4	(-15, -6.5)
			IKDC	Meaningful improvement (≥ 7&≤19)	44	-13.5	(-16.2, -10.6)
		6 months	KOOS-12 Total	Meaningful improvement (≥ 2&≤15)	37	-11.7	(-15.3, -8.3)
			IKDC	Meaningful improvement (≥ 7&≤19)	40	-16.7	(-20.3, -13.4)
	Control (autoserum)	3 months	KOOS-12 Total	Meaningful improvement (≥ 2&≤15)	52	-6.8	(-9.4, -4.2)
			IKDC	Meaningful improvement (≥ 7&≤19)	38	-11.1	(-13.6, -8.7)
		6 months	KOOS-12 Total	Meaningful improvement (≥ 2&≤15)	53	-4.6	(-7.9, -1.8)
			IKDC	Meaningful improvement (≥ 7&≤19)	35	-9.3	(-12.3, -6.2)
WOMAC Function	ADMSCs injection	3 months	KOOS-12 Function	Meaningful improvement (≥ 2&≤15)	41	-7.6	(-10.4, -4.9)
			IKDC	Meaningful improvement (≥ 7&≤19)	44	-9.7	(-11.8, -7.6)
			SF-36 Physical	Meaningful improvement (≥ 1&≤5)	6	-8.3	(-13, -3.3)
		6 months	KOOS-12 Function	Meaningful improvement (≥ 2&≤15)	36	-9.5	(-12.3, -7)
			IKDC	Meaningful improvement (≥ 7&≤19)	40	-12.1	(-14.6, -9.7)
			SF-36 Physical	Meaningful improvement (≥ 1&≤5)	9	-9.6	(-15.8, -3)
	Control (autoserum)	3 months	KOOS-12 Function	Meaningful improvement (≥ 2&≤15)	45	-6.8	(-9.4, -4.2)
			IKDC	Meaningful improvement (≥ 7&≤19)	38	-11.1	(-13.6, -8.7)
			SF-36 Physical	Meaningful improvement (≥ 1&≤5)	18	-4.7	(-6.6, -2.7)
		6 months	KOOS-12 Function	Meaningful improvement (≥ 2&≤15)	31	-3.9	(-6, -1.7)
			IKDC	Meaningful improvement (≥ 7&≤19)	35	-6.7	(-8.8, -4.5)
			SF-36 Physical	Meaningful improvement (≥ 1&≤5)	14	-5.0	(-8.4, -1.7)
VAS Pain	ADMSCs injection	3 months	KOOS-12 Pain	Meaningful improvement (≥ 2&≤17)	35	-13.7	(-19.6, -7.7)
		6 months	KOOS-12 Pain	Meaningful improvement (≥ 2&≤17)	35	-13.3	(-19.7, -8.1)
	Control (autoserum)	3 months	KOOS-12 Pain	Meaningful improvement (≥ 2&≤17)	33	-7.6	(-11.9, -3.1)
		6 months	KOOS-12 Pain	Meaningful improvement (≥ 2&≤17)	38	-7.7	(-12.2, -3.7)

Note. WOMAC and VAS were scored such that higher values indicated worse function and pain; therefore, negative change scores represent improvement

**Fig. 1:**
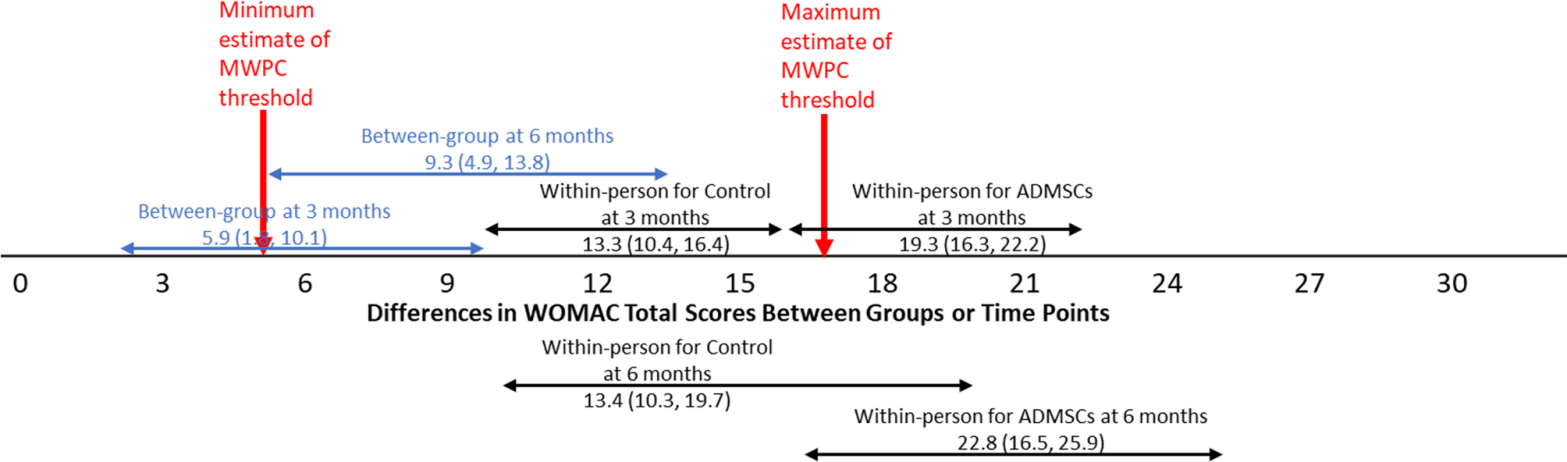
Estimated between-group differences in adjusted* means (with 95% confidence intervals) and adjusted* within-person change scores of each group at each time point for WOMAC Total. Note. * denotes adjusted means controlling for baseline scores

**Fig. 2:**
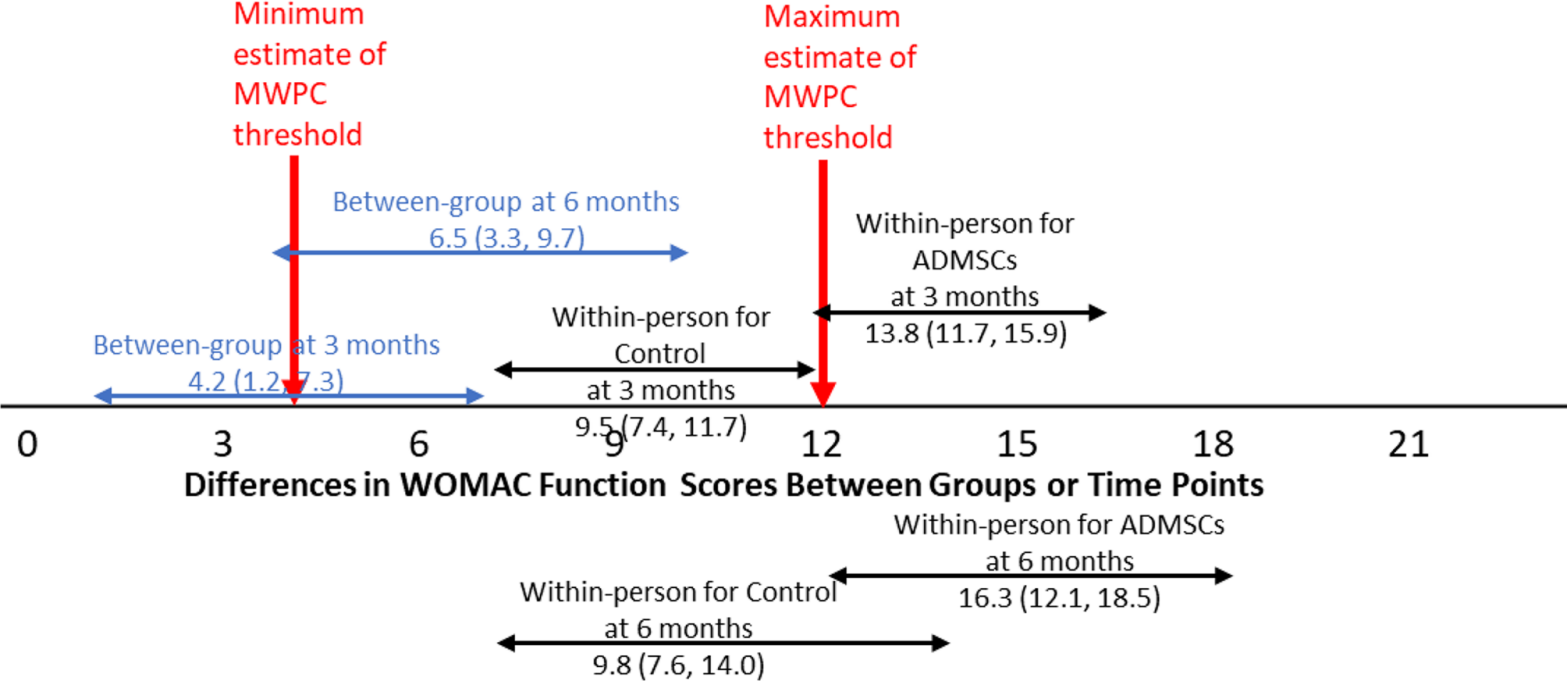
Estimated between-group differences in adjusted* means (with 95% confidence intervals) and adjusted* within-person change scores of each group (with 95% confidence intervals) at each time point for WOMAC Function. Note. * denotes adjusted means controlling for baseline scores

**Fig. 3:**
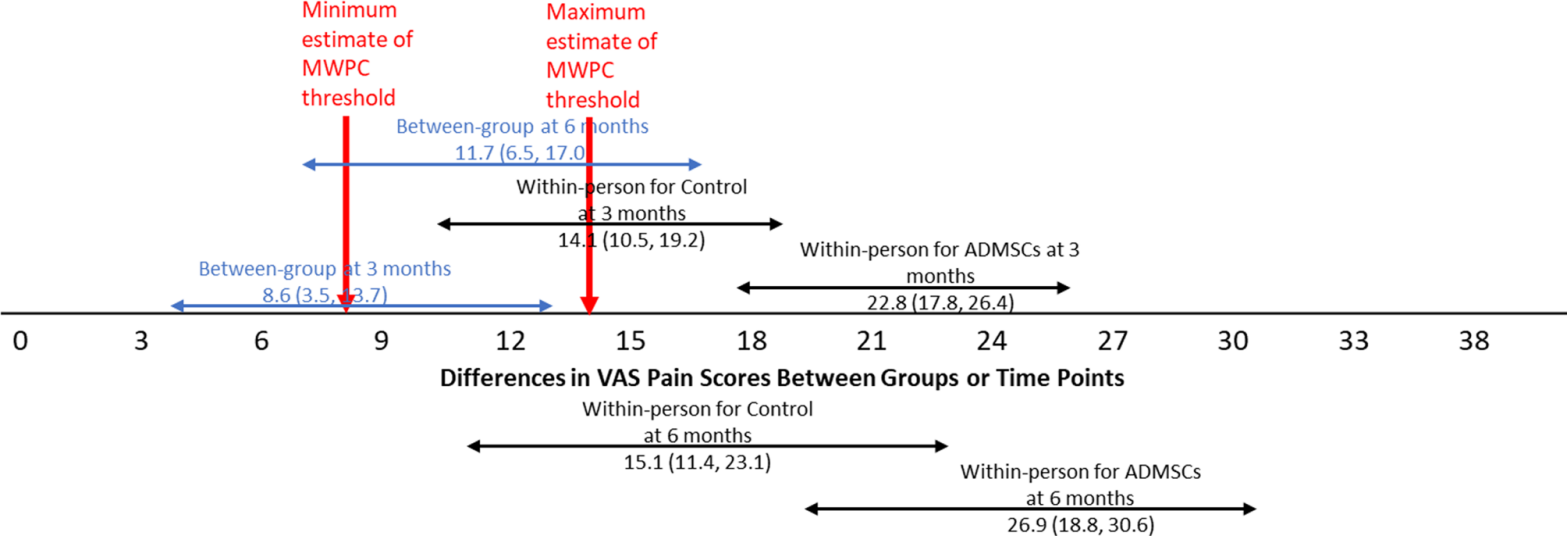
Estimated between-group differences in adjusted* means (with 95% confidence intervals) and adjusted* within-person change scores of each group (with 95% confidence intervals) at each time point for VAS Pain. Note. * denotes adjusted means controlling for baseline scores

Between-group differences in adjusted means (Table 2) showed significantly better scores in the ADMSCs injection group compared to the Control (autoserum) group at both time points across all measures, suggesting patients in the ADMSCs injection groups showed significantly more improvement. Additionally, a larger group difference was observed at 6 months than 3 months. Figures 1, 2 and 3 show that the between-group difference at 3 months was below the lower bound of the proposed MWPC threshold, whereas the between-group difference at 6 months fell within the MWPC range, with confidence intervals fully overlapping with the MWPC range.

**Table 2:** Estimated difference in adjusted* means between the ADMSCs injection and control (autoserum) groups at 3 months and 6 months

Time	Measure	Adjusted group differences	Confidence Interval	Degree of Freedom	*P*-value
3 months	WOMAC Total	5.9	(1.8, 10.1)	243	< 0.01
	WOMAC Function	4.2	(1.2, 7.3)	243	< 0.01
	VAS Pain	8.6	(3.5, 13.7)	243	< 0.01
6 months	WOMAC Total	9.3	(4.9, 13.8)	244	< 0.001
	WOMAC Function	6.5	(3.3, 9.7)	244	< 0.001
	VAS Pain	11.7	(6.5, 17.0)	244	< 0.001

Note. * denotes adjusted means controlling for baseline scores. Adjusted group differences were calculated using Control (autoserum) minus ADMSCs injection. WOMAC and VAS were scored such that higher values indicated worse function and pain; therefore, positive group differences indicate that the Control (autoserum) group reported worse function and pain than the ADMSCs injection group

Within-person adjusted mean change scores (Table 3) increased over time in both groups, with significantly greater improvement in the ADMSC group, particularly at 6 months. Figures 1, 2 and 3 present the adjusted mean change scores with 95% confidence intervals for both groups at both time points, displayed alongside the proposed MWPC threshold range. The mean change scores of the ADMSC injection group at both time points, along with their confidence intervals, all exceed the upper bound of the MWPC threshold range across all three focal measures, indicating meaningful improvement. For the Control (autoserum) group, the mean change scores were generally smaller and often remained within or near the MWPC range.

**Table 3:** Adjusted* within-person change scores of each group at 3 months, 6 months, and 3 years

Measure	Time	ADMSCs injection		Control (autoserum)		*P*-value
		Mean Change	Confidence Interval	Mean Change	Confidence Interval	
WOMAC Total	3 months	-19.3	(-22.2, -16.3)	-13.3	(-16.4, -10.4)	< 0.01
	6 months	-22.8	(-25.9, -16.5)	-13.4	(-19.7, -10.3)	< 0.01
WOMAC Function	3 months	-13.8	(-15.9, -11.7)	-9.5	(-11.7, -7.4)	< 0.01
	6 months	-16.3	(-18.5, -12.1)	-9.8	(-14, -7.6)	< 0.01
VAS Pain	3 months	-22.8	(-26.4, -17.8)	-14.1	(-19.2, -10.5)	< 0.01
	6 months	-26.9	(-30.6, -18.8)	-15.1	(-23.1, -11.4)	< 0.01

Note. * denotes adjusted means controlling for baseline scores. WOMAC and VAS were scored such that higher values indicated worse function and pain; therefore, negative change scores represent improvement

## Discussion

The present study applies an advanced method of predictive modeling to estimate the MWPC thresholds for the measures of WOMAC Total, WOMAC PF, and VAS Pain among knee OA patients using clinical trial data. We proposed a range of MWPC thresholds: 5 to 17 score points in WOMAC Total on a scale of 0-100, 4 to 12 score points in WOMAC PF on a scale of 0-100, 8 to 14 for in VAS Pain on a scale of 0-100. These threshold ranges were estimated between the meaningfully improved group and non-improved group based on the predictive modeling approach, which adjusted for the unequal sample sizes of the two groups to increase the robustness of the MWPC estimates [14]. Furthermore, these estimates were derived from longitudinal within-person change data by combining the MWPC values of both treatment and control groups at two follow-up time points. Hence, this proposed range is applicable to data with or without treatment and at the two commonly used time points.

The estimated values for MWPC produced in this study are in a wider range than those reported in previous studies [5, 10, 15] mentioned in the introduction for WOMAC PF and VAS Pain. This is not unexpected because the mean change method was used in previous studies, whereas we used the predictive modeling approach. We considered MWPC thresholds in both treatment and control groups at two time points, leading to a wider MWPC range that can be applicable for diverse contexts. Moreover, the patient groups are not the same. The present study also calculates the MWPC range for WOMAC Total, which was not explored by previous studies.

This study also showcased how the proposed MWPC range was applied to interpret the meaningfulness of treatment benefits. We found that after 6 months, patients who received the ADMSCs injection showed significantly greater improvement than patients in the Control (autoserum) group across all three focal measures. This improvement fell within the proposed MWPC range, although a small portion of the confidence interval extended below the lower bound. This suggests that the average treatment effect corresponds to a meaningful improvement in patients’ physical function and pain, as many patients would consider it significant, though a small portion might perceive the treatment effect as less meaningful. Additionally, the comparison between the proposed MWPC range and the within-person change scores strongly suggests that, on average, patients in the ADMSCs injection group experience meaningful improvement. In contrast, patients in the control group experience less meaningful improvement.

The MWPC thresholds provide a clinically useful range that healthcare stakeholders can use to interpret whether patients have achieved meaningful improvement. In practice, the lower bound of the range can serve as a conservative benchmark to identify meaningful improvement, while the upper bound can indicate more substantial or highly confident changes, allowing flexibility in applying these thresholds based on clinical judgment, patient goals, treatment costs, and other factors.

This study has several limitations. First, due to the small number of patients who reported worsened physical function or pain, we did not calculate or evaluate the MWPC thresholds for the worsening group separately. Future research can be conducted if a worsened group with an appropriate sample size is observed. Second, we used KOOS, IKDC, and SF-36 as anchors rather than PGIC because PGIC was not included in the data collection. This approach may introduce some uncertainty, as the relationship between these measures and PGIC can vary across different populations, interventions, and study settings. Nevertheless, leveraging multiple PROMs as anchors remains a practical and clinically relevant strategy, especially when direct PGIC data are unavailable. In our study, the consistency of MWPC thresholds across multiple anchors supports the robustness of the proposed MWPC thresholds despite this limitation. Third, we selected anchor measures that are commonly used to evaluate physical function and pain. Future research could explore the use of knee OA-specific measures as anchor measures.

In conclusion, this study proposes MWPC thresholds for WOMAC Total, WOMAC PF, and VAS pain for patients with knee OA based on the predictive modeling approach. Using the proposed MWPC range as a reference, patients in the treatment group showed meaningful improvement compared to the control group, and the within-person change scores, on average, indicated meaningful improvement for patients in the treatment group. In sum, the proposed MWPC thresholds could be beneficial to a variety of stakeholders in clinical practice, research, and regulatory settings as a tool to identify meaningful change in physical function and pain in response to treatment and evaluate the clinical meaningfulness of treatment benefits.

## Supplementary Information

Below is the link to the electronic supplementary material.

Supplementary Material 1

## Data Availability

The datasets generated and/or analysed during the current study are not publicly available due to clinical trial data but are available from the corresponding author on reasonable request.
