# Supplementary material for: Meaningful changes in physical function and pain in patients with knee osteoarthritis
Source: J Patient Rep Outcomes. 2025 Sep 26;9:112. doi: 10.1186/s41687-025-00941-x (PMC12474783; doi:10.1186/s41687-025-00941-x)
Supplement: Supplementary file 1 — Supplementary Material 1 [file 41687_2025_941_MOESM1_ESM.docx]

Appendix. Correlations of mean change scores between anchor measures and focal measures.

Table 1. Anchor change categories to examine MWPC in WOMAC Total.

| KOOS-12 Total (r_w12_ = 0.65*; r_w24_ = 0.71*) | | 3-month | 12-month |
| --- | --- | --- | --- |
|  | Better | 15.1 | 16.8 |
|  | Somewhat better | 4.7 | 4.0 |
|  | Very small improvement | -1.6 | -2.5 |
|  | Same | -1.0 | -2.6 |
|  | Very small deterioration | -3.8 | -7.7 |
|  | Somewhat worse | -12.8 | -12.8 |
|  | Worse | -12.6 | -32.9 |
| IKDC (r_w12_ =-0.57; r_w24_ =-0.60) | | 3-month |  |
|  | Very much improved | 19.3 |  |
|  | Much improved | 13.0 |  |
|  | Minimally Improved | 7.1 |  |
|  | No change | -3.6 |  |
|  | Minimally worse | -5.4 |  |
|  | Much worse | -2.9 |  |

Note. *calculated based on raw summed KOOS-12 total scores. r refers to Pearson’s correlation.

Table 2. Anchor change categories to examine MWPC in WOMAC PF.

| KOOS-12 Function (r_w12_ = 0.58*; r_w24_ = 0.66*) | | 3-month | 12-month |
| --- | --- | --- | --- |
|  | Better | 15.9 | 17.0 |
|  | Somewhat better | 3.6 | 3.6 |
|  | Very small improvement | -0.4 | -3.8 |
|  | Same | 5.0 | 4.7 |
|  | Very small deterioration | -11.5 | -12.5 |
|  | Somewhat worse | -19.3 | -22.5 |
|  | Worse | -14.2 | -21.1 |
| SF-36 PF (r_w12_ = -0.46; r_w24_ = -0.56) | | 6-month | 12-month |
|  | Lot better | 4.08 | 4.99 |
|  | Little better | -0.58 | 0.32 |
|  | Same | 0.89 | 0.46 |
|  | Little worse | -2.34 | -3.86 |
|  | Lot worse | -3.47 | -4.74 |
| IKDC (r_w12_ = -0.56; r_w24_ = -0.58) | | 3-month |  |
|  | Very much improved | 19.3 |  |
|  | Much improved | 13.0 |  |
|  | Minimally Improved | 7.1 |  |
|  | No change | -3.6 |  |
|  | Minimally worse | -5.4 |  |
|  | Much worse | -2.9 |  |

Note. *calculated based on raw summed KOOS-12 function scores. r refers to Pearson’s correlation.

Table 3. Anchor change categories to examine MWPC in VAS Pain.

| KOOS-12 Pain (r_w12_ = 0.50*; r_w24_ = 0.44*) | | 3-month | 12-month |
| --- | --- | --- | --- |
|  | Better | 17.1 | 18.8 |
|  | Somewhat better | 5.5 | 5.4 |
|  | Very small improvement | -1.3 | -2.1 |
|  | Same | 0.3 | -1.1 |
|  | Very small deterioration | -4.8 | -7.8 |
|  | Somewhat worse | -14.6 | -10.8 |
|  | Worse | -12.1 | -33.2 |

Note. *calculated based on raw summed KOOS-12 pain scores. r refers to Pearson’s correlation.
